# Development of a new dipstick (Cholkit) for rapid detection of *Vibrio cholerae* O1 in acute watery diarrheal stools

**DOI:** 10.1371/journal.pntd.0006286

**Published:** 2018-03-14

**Authors:** Md. Abu Sayeed, Kamrul Islam, Motaher Hossain, Noor Jahan Akter, Md. Nur Alam, Nishat Sultana, Farhana Khanam, Meagan Kelly, Richelle C. Charles, Pavol Kováč, Peng Xu, Jason R. Andrews, Stephen B. Calderwood, Jakia Amin, Edward T. Ryan, Firdausi Qadri

**Affiliations:** 1 Infectious Diseases Division, International Centre for Diarrhoeal Disease Research, Bangladesh (icddr,b), Dhaka, Bangladesh; 2 Incepta Pharmaceuticals Ltd, Savar, Dhaka, Bangladesh; 3 Division of Infectious Diseases, Massachusetts General Hospital, Boston, Massachusetts, United States of America; 4 Department of Medicine, Harvard Medical School, Boston, Massachusetts, United States of America; 5 National Institute of Diabetes, Digestive and Kidney Diseases (NIDDK), Laboratory of Bioorganic Chemistry (LBC), National Institutes of Health, Bethesda, Maryland, United States of America; 6 Division of Infectious Diseases and Geographic Medicine, Stanford University School of Medicine, Stanford, California, United States of America; 7 Department of Microbiology and Immunobiology, Harvard Medical School, Boston, Massachusetts, United States of America; 8 Department of Immunology and Infectious Diseases, Harvard T.H. Chan School of Public Health, Boston, Massachusetts, United States of America; University of California San Diego School of Medicine, UNITED STATES

## Abstract

Recognizing cholera cases early, especially in the initial phase of an outbreak and in areas where cholera has not previously circulated, is a high public health priority. Laboratory capacity in such settings is often limited. To address this, we have developed a rapid diagnostic test (RDT) termed Cholkit that is based on an immunochromatographic lateral flow assay for the diagnosis of cholera cases using stool. Cholkit contains a monoclonal antibody (ICL-33) to the O-specific polysaccharide (OSP) component of *V*. *cholerae* O1 lipopolysaccharide, and recognizes both Inaba and Ogawa serotypes. We tested the Cholkit dipstick using fresh stool specimens of 76 adults and children presenting with acute watery diarrhea at the icddr,b hospital in Dhaka, Bangladesh. We compared Cholkit’s performance with those of microbial culture, PCR (targeting the *rfb* and *ctxA* genes of *V*. *cholerae*) and the commercially available RDT, Crystal VC (Span Diagnostics; Surat, India). We found that all stool specimens with a positive culture for *V*. *cholerae* O1 (n = 19) were positive by Cholkit as well as Crystal VC. We then used Bayesian latent class modeling to estimate the sensitivity and specificity of each diagnostic assay. The sensitivity of Cholkit, microbiological culture, PCR and Crystal VC was 98% (95% CI: 88–100), 71% (95% CI: 59–81), 74% (95% CI: 59–86) and 98% (95% CI: 88–100), respectively. The specificity for *V*. *cholerae* O1 was 97% (95% CI: 89–100), 100%, 97% (95% CI: 93–99) and 98% (95% CI: 92–100), respectively. Of note, two Crystal VC dipsticks were positive for *V*. *cholerae* O139 but negative by culture and PCR in this area without known circulating epidemic *V*. *cholerae* O139. In conclusion, the Cholkit dipstick is simple to use, requires no dedicated laboratory capacity, and has a sensitivity and specificity for *V*. *cholerae* O1 of 98% and 97%, respectively. Cholkit warrants further evaluation in other settings.

## Author summary

Cholera is a severely dehydrating diarrheal disease that can lead to death if remains untreated. The incidence of case fatality is higher at the beginning of the outbreak. Diagnosis of cholera in the early stage of outbreak is a high public health priority. Although countries facing complex emergencies are more vulnerable to cholera outbreak, laboratory capacity in such settings is usually limited. To address this, here we report the development of a rapid diagnostic test (RDT) termed Cholkit for the diagnosis of cholera cases using stool and the assessment of its performance with those of microbial culture, PCR and Crystal VC assay, a commercially available dipstick using a latent class modeling approach.

## Introduction

Cholera is an acute watery diarrheal disease caused mainly by *Vibrio cholerae* serogroup O1 and less commonly by *V*. *cholerae* O139. Cholera can lead to severe diarrhea and death if untreated. *V*. *cholerae* O1 is transmitted through fecal-oral contamination, and cholera is thus predominantly associated with lack of safe drinking water, proper sanitation and personal hygiene. Cholera is an important public health problem in many parts of Asia, Africa and Latin America [1–3]. Globally, 3–5 million cases and over 100,000 deaths occur annually due to cholera [4]. Countries facing complex emergencies are more vulnerable to cholera outbreaks [5]. The case fatality rate is often highest at the beginning of an outbreak, and delayed recognition of a cholera outbreak often results in a delayed public health response that can result in high morbidity and mortality [6]. Thus, the rapid and correct detection of cholera cases in the initial stages of an outbreak is critical. Patients with cholera often present with acute watery diarrhea, and although rapid presentation of multiple individuals with severe dehydration, especially adults, is highly suggestive of a cholera outbreak, a firm diagnosis is critical to initiating appropriate public health responses and communications [7–9]. Unfortunately, populations at highest risk for cholera are usually poorly supported by diagnostic capacity: laboratory facilities are usually rudimentary or totally absent, and trained health personnel are often not available. In such settings, there is a pressing need for simple and inexpensive rapid diagnostic tests to correctly identify patients with cholera. Here, we report the development of a new rapid diagnostic dip-stick test, Cholkit, that can be used to evaluate stool samples in suspected cholera patients. This assay is based on the detection of *V*. *cholerae* O1 lipopolysaccharide (LPS) in stool, and here, we report our analysis of Cholkit’s performance among patients with acute watery diarrhea in Dhaka, Bangladesh using a latent class modeling approach, comparing its performance to those of microbial culture, PCR (assessing *V*. *cholerae* O1 and O139-specific *rfb* genes and cholera toxin gene *ctxA*) analysis of stool, and Crystal VC assay, a commercially available dipstick designed to detect both *V*. *cholerae* O1 and O139.

## Methods and materials

### Ethics statement

This study was approved by the Research Review and the Ethical Review Committees of the International Centre for Diarrhoeal Disease Research, Bangladesh (icddr,b) and the Institutional Review Board (IRB) of the Massachusetts General Hospital. Written consent was obtained from the guardians of children (1–17 years) as well as assent from those 11–17 years of age; adult participants (18–59 years) provided their own consent.

### Study participants and specimen collection

We collected stool from 76 hospitalized adults and children at the Dhaka Hospital of the icddr,b who presented with acute watery diarrhea.

### Microbiological culture of stool

We performed conventional stool culture by streaking stool directly on selective TTGA (taurocholate-tellurite gelatin agar) plates, and incubated these plates overnight at 37°C. Fecal specimens were concurrently enriched overnight at 37°C in alkaline peptone water (1% peptone, 1% NaCl; pH- 8.5), followed by plating on TTGA to isolate *V*. *cholerae*. Colonies morphologically consistent with *V*. *cholerae* were analyzed by slide agglutination with monoclonal antibodies specific to *V*. *cholerae* serovar O1 (Ogawa or Inaba) and O139 [10, 11].

### Crystal VC dipstick test

The Crystal VC test was performed on fresh samples of stool according to the manufacturer’s instructions. Briefly, two drops of liquid stool were added into the sample processing vial and mixed gently. Four drops of the processed sample were then put in a test tube. The Crystal VC test strip was dipped into the tube and the results were interpreted according to the manufacturer’s protocol.

### PCR

Two ml of watery stool were spun at 10,000 rpm for 10 minutes. The pellet was resuspended in 200 μl of phosphate buffered saline (PBS) and used for DNA extraction with the QiaAmp stool DNA extraction kit (Qiagen) following the manufacturer’s instructions. Multiplex PCR assays were performed on a Thermo cycler C-1000 instrument (Bio-Rad). *V*. *cholerae* O1-*rfb* specific primers: O1-F(5´-GTTTCACTGAACAGATGGG-3´),O1-R(5´-GGTCATCTGTAAGTACAAC-3´); *V*. *cholerae* O139-*rfb* specific primers O139-F (5´-AGCCTCTTTATTACGGGTGG-3´), O139-R (5´-GTCAAACCCGATCGTAAAGG-3´); and cholera toxin gene primers: *ctxA*-F (5´-CTCAGACGGGATTTGTTAGGC-3´), *ctxA*-R (5´-TCTATCTCTGTAGCCCCTATTA-3´) were used to amplify O1 *rfb* (amplicon size 192 bp), O139 *rfb* (amplicon size 449 bp) and *ctxA* (amplicon size 302 bp) genes, respectively, using previously described procedures [10, 12]. PCR products were analyzed on a 1% agarose gel using Gel Red (BioTium, USA) stain.

### Production of anti-*V*. *cholerae* LPS antibody in murine ascites fluid

A previously isolated and characterized monoclonal antibody, ICL33 generated at the icddr,b was used for preparing the dipstick. The procedure for isolating the monoclonal antibody involved use of female BALB/c mice that were immunized with an acetone extract of *V*. *cholerae* O1 Inaba strain T-19479 (50 μg per dose) four times at weekly intervals [13]. The first dose was administered subcutaneously with Freundʹs complete adjuvant. Subsequent doses were given intraperitoneally with Freundʹs incomplete adjuvant. Four days after the last dose, spleen cells from two immunized BALB/c mice were fused with SP2/0 myeloma cells [13, 14]. After screening the reactivity of supernatant fluids harvested from the hybridomas against an acetone extract of *V*. *cholerae* 01 Inaba strain T-19479 and LPS isolated from *V*. *cholerae* O1 Inaba strain T-19479 and Ogawa strain X25049 by ELISA, we selected one reactive hybridoma that was cloned twice by limiting dilution. The monoclonal antibody (ICL-33) secreted by this clone was of IgG3 isotype and specific to LPS of both *V*. *cholerae* O1 Inaba and Ogawa. This clone was used to make ascites fluid containing anti-*V*. *cholerae* O1 LPS antibody using a previously described procedure [13]. In brief, six to eight week old BALB/c mice (n = 23) were primed with pristane (Sigma). These pristane-primed mice were then injected intraperitoneally with this clone (1.5 x 10^6^ to 2.0 x 10^6^ cells /mouse). Ascites fluid was formed 10–14 days after the injection of the cell line. After collection of ascites fluid, it was heated at 56°C for 30 min. The heat inactivated fluid was centrifuged at 3000 rpm for 10 min at 20°C. The supernatant was then separated, followed by filtration with 0.45μm and 0.2μm filters (Sartorius, Germany) respectively.

### Purification of monoclonal IgG3 antibody from ascites

Protein G GraviTrap (GE Healthcare Life Sciences) was used to purify the murine IgG3 monoclonal antibody (ICL-33) targeting *V*. *cholerae* LPS from ascites following the standard procedure recommended by the manufacturer. Briefly, the affinity column was equilibrated with 1× binding buffer, and the ascites sample (diluted 2.5 times with 1× binding buffer) was then applied. After washing the column with binding buffer, the monoclonal antibody was eluted into falcon tubes containing neutralizing buffer using 1× elution buffer; the protein concentration of the recovered antibody was determined by Bio-Rad protein assay.

### Detection of *V*. *cholerae* LPS and OSP-specific antibody responses

We confirmed anti-LPS and OSP IgG specificity of monoclonal antibody, ICL-33 using standard enzyme-linked immunosorbent assay (ELISA) protocols [15, 16]. Briefly, we coated ELISA plates with *V*. *cholerae* O1 Inaba and Ogawa LPS (2.5 μg/mL) and OSP:BSA (1 μg/mL) in PBS [15, 16]. Reagents were produced as previously described [17, 18]. To each well, we added 100 μL of purified monoclonal antibody (1,000, 10,000, 100,000 and 1,000,000 dilutions in 0.1% BSA in phosphate buffered saline-Tween; the initial antibody concentration was 1.14 μg/mL), and detected the presence of antigen-specific antibodies using horseradish peroxidase-conjugated anti-mouse IgG antibody (diluted 1:1000 in 0.1% BSA in phosphate buffered saline-Tween) (Southern Biotech, Birmingham, AL). After 1.5 h incubation at 37°C, we developed the plates with a 0.55 mg/mL solution of 2,2ʹ-azino-bis (3-ethylbenzothiazoline-6-sulfonic acid) (ABTS; Sigma) with 0.03% H_2_O_2_ (Sigma), and determined the optical density at 405 nm with a Vmax microplate kinetic reader (Molecular Devices Corp. Sunnyvale, CA). Plates were read for 5 min at 30 s intervals, and the results were reported as millioptical density units per minute (mOD/min).

### Slide agglutination test

The ability of the ICL-33 monoclonal antibody to detect *V*. *cholerae* O1 strains was further assessed using a slide agglutination test with TTGA-grown *V*. *cholerae* bacteria. *V*. *cholerae* Ogawa strain X25049, *V*. *cholerae* Inaba strain T-19479 and *V*. *cholerae* O139 strain 134B were cultured on TTGA plate at 37°C for overnight. Bacteria from a single colony were added with 10 μl of monoclonal antibody at different dilutions on a glass slide for agglutination. The appearance of agglutination within 2 minutes was considered a positive reaction [13].

### Gold preparation and conjugation

We prepared 20 nm colloidal gold by adding 0.01% HAuCl_4_ with 0.024% sodium citrate and boiled the solution until it became a red wine color [19]. The colloidal gold was then filtered through a 0.2 μm filter. We adjusted the pH of the gold solution to 9.5 (optimum pH for conjugation) and added 18 μg of purified ICL-33 monoclonal antibody to conjugate with 1 ml of the colloidal gold (minimal concentration for conjugation) [19]. We then added 20% BSA to block non-specific binding sites. Monoclonal antibody conjugated to gold was then centrifuged at 10000 rpm for 45 min at 4°C. The supernatant was discarded and the pellet was re-suspended in 0.02M Tris buffer containing 1% BSA.

### Preparation of a lateral flow strip (Cholkit)

We used a baking card containing a nitrocellulose membrane on which ICL-33 monoclonal antibody (test line) and goat anti-mouse IgG (control line) were dispensed in two lines, respectively by using a Rapid test dispenser (HM3030). The dispensed membrane was dried for 1 hour 30 min followed by blocking with 1% BSA-PBS for 20 min. Conjugate pads were made by soaking glass fiber in gold-monoclonal antibody conjugate solution and drying for 2 hours, and then pasting on the baking card in a way that overlapped the nitrocellulose membrane (High flow plus 120 Membrane card). The sample pad (glass fiber) was placed at the bottom of the backing card to overlap with the conjugate pad to facilitate the flow of sample from sample vial to strip. To accelerate the migration of the sample through the strip, we used cellulose fiber as an absorbent pad and pasted on the baking card opposite to the conjugate pad. All pads were cut to make the required shape by using a Guillotine cutter (CT300 and ZQ2000).

### Cholkit test

**Fig 1:**
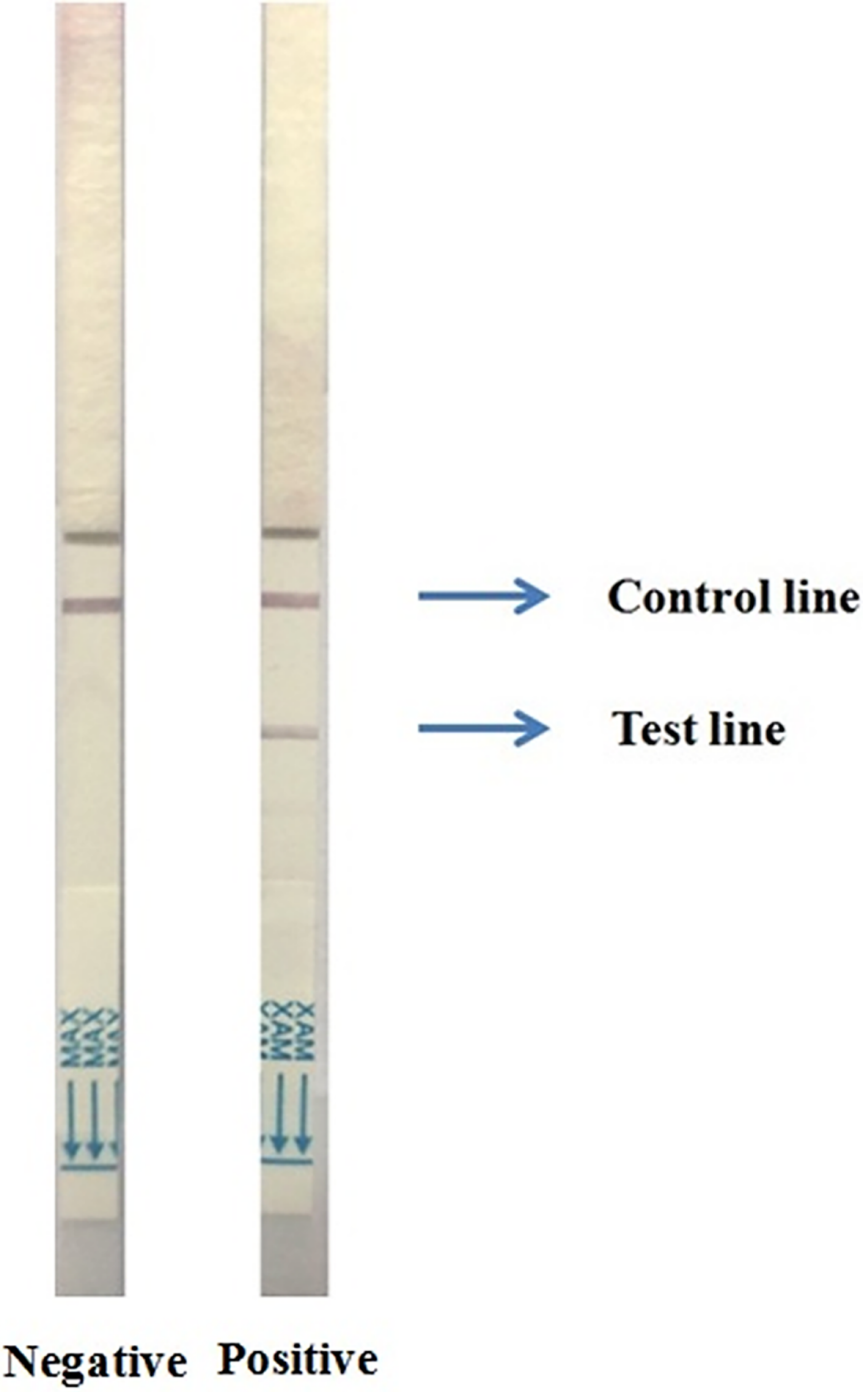
Two Cholkit dipsticks showing characteristic negative (left image) and positive (right image) results after 15 min sample run.

We diluted 5 drops of watery stool with Tris-NaCl-Tween at a 1:1 dilution in a microcentrifuge tube and dipped the Cholkit strip into it for 15 min; the test line and/or control line appeared as a red color. Appearance of both lines indicated that the sample was positive for *V*. *cholerae* O1; appearance of only the control line but not the test line indicated a negative result for the test (Fig 1).

### Statistical analysis

We used Graphpad Prism4 for data management, analysis, and graphical presentation. Sensitivity and specificity of different diagnostic tests were calculated using latent class modeling.

### Latent class modeling

We estimated the sensitivity and specificity of each of the diagnostic tests using a Bayesian framework with latent class models [20]. For prior information, we assumed that the sensitivity of culture was 60–90% and specificity was 99.99–100% [21]. We used broader prior estimates of sensitivity and specificity for PCR, Crystal VC and Cholkit. The prior assumed sensitivity and specificity for PCR were 50–100% and 90–100%, respectively. The prior estimates of sensitivity and specificity for both Crystal VC and Cholkit were 0–100%. We used a Gibbs sampler with 100,000 iterations to generate posterior estimates with 95% credible intervals (CI) for sensitivity and specificity; all analyses were performed using Python.

## Results

### Characteristics of study participants

Characteristics of all study participants are presented in Table 1. Of 76 patients who were enrolled as study participants, 45 (59%) were male. The median age was 26 years with a range of 5 months to 60 years. Out of all the study patients, 62% presented to the icddr, b with severe dehydration and 29% with moderate dehydration.

**Table 1:** Characteristics of study participants.

Parameters	Values
Median age in year (25^th^ and 75^th^ percentiles)	26 (17, 39)
No. of males (%)	45 (59)
Patients with severe dehydration (%)	47 (62)
Patients with moderate dehydration (%)	22 (29)
Patients with no dehydration (%)	7 (9)

### The monoclonal antibody (mAb) used in the Cholkit RDT recognizes both Inaba and Ogawa, *V*. *cholerae* O1 OSP, LPS and *V*. *cholerae* O1 organisms, but not *V*. *cholerae* O139

**Fig 2:**
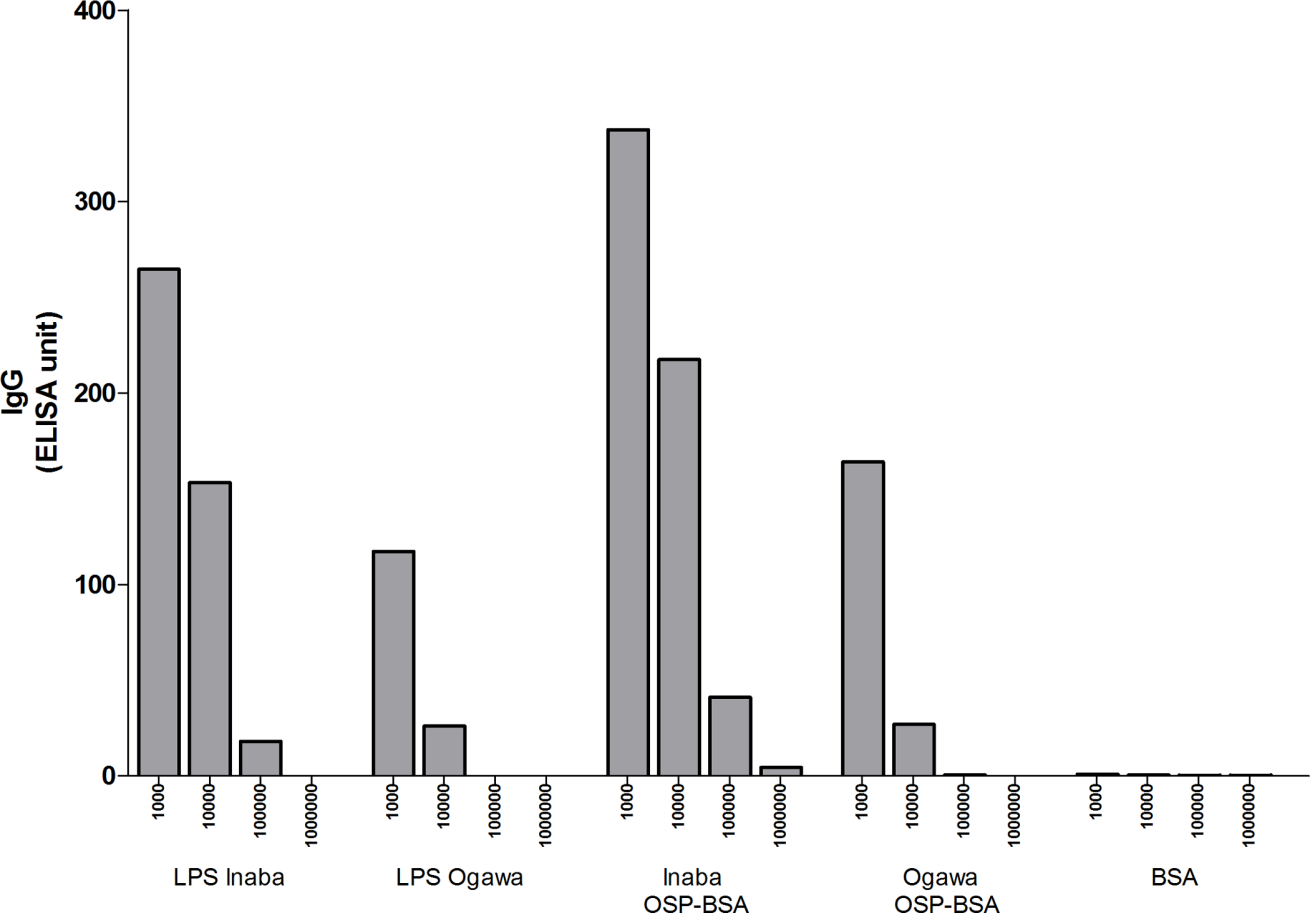
Immunoreactivity of the ICL-33 monoclonal antibody at 1:1,000 (114 ng); 1:10,000 (11.4 ng); 1:100,000 (1.14 ng) and 1:1000,000 (114 pg) dilutions to purified lipopolysaccharide (LPS), O-specific polysaccharide (OSP) components of *V*. *choleare* O1 Ogawa and Inaba serotypes and BSA: Bovine serum albumin.

The monoclonal antibody used in the Cholkit assay was highly reactive with purified *V*. *cholerae* O1 Inaba and Ogawa OSP, as well as LPS, with reactivity detectable to the 1.14 ng antibody level for Inaba *V*. *cholerae* O1 OSP, and 114 ng for Ogawa *V*. *cholerae* O1 OSP (Fig 2). The monoclonal antibody showed characteristic agglutination reaction with both *V*. *cholerae* O1 Ogawa and Inaba strains, but no agglutination was found for *V*. *cholerae* O139 when slide agglutination test was performed.

### Comparison of stool culture, Cholkit, Crystal VC and PCR

**Fig 3:**
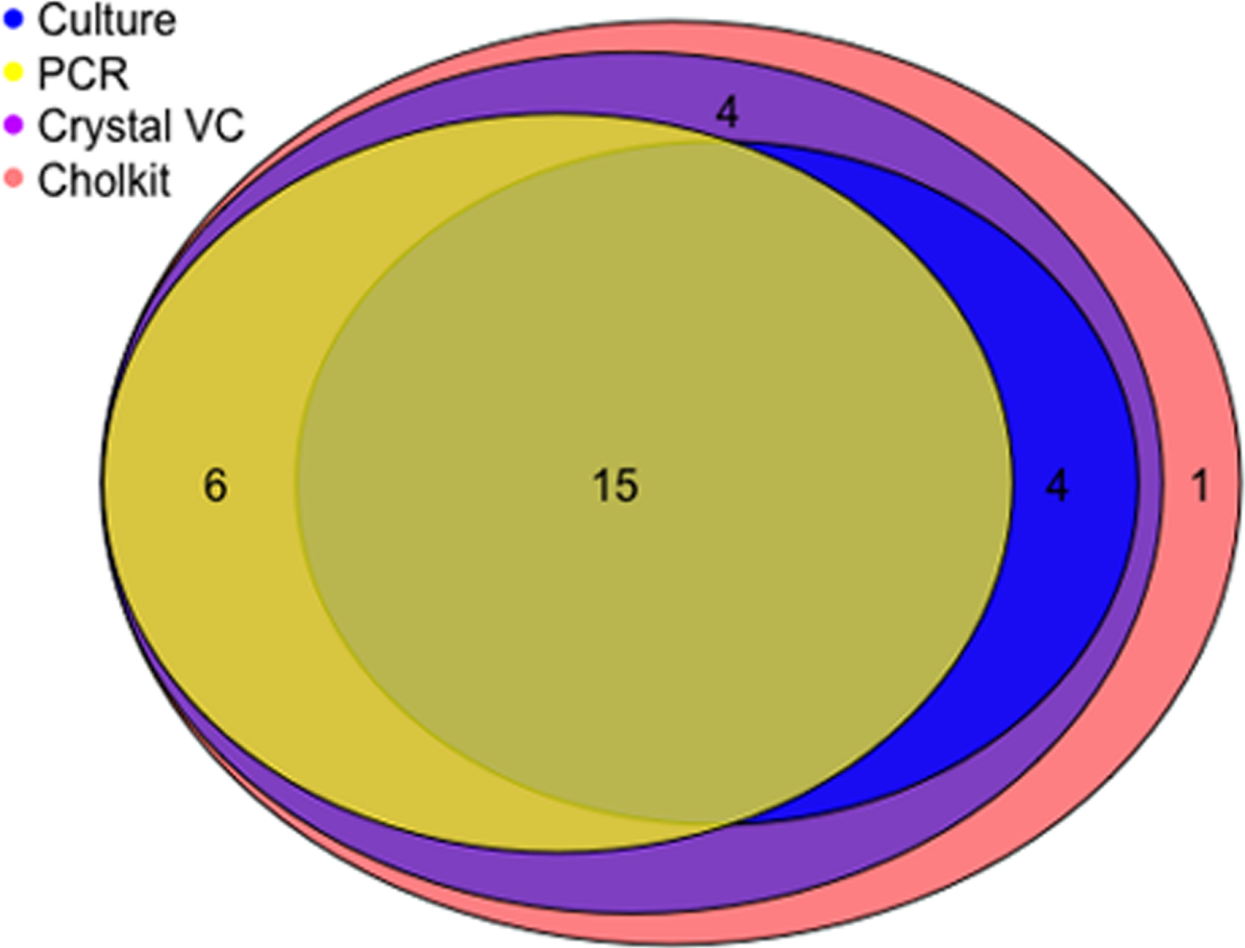
Euler diagram that illustrates overlap of the positive test results from four diagnostic tests analyzed in the Bayesian latent class modeling.

Stool specimens from 76 patients were tested by all four of microbial culture, Cholkit, Crystal VC and PCR assays. Nineteen samples were positive by culture and all of them were confirmed as positive for *V*. *cholerae* O1 Inaba except one sample that was *V*. *cholerae* O1 Ogawa. Out of 19 stools positive by culture, all 19 (100%) were positive by both Cholkit and Crystal VC assays, and 15 (79%) were positive by PCR (Table 2 and Fig 3). Of all patients with a negative stool culture (n = 57), Cholkit, Crystal VC and PCR were positive for 11 (19%), 12 (21%), and 6 (11%), respectively. Six specimens that were culture negative but PCR positive were also positive by both Cholkit and Crystal VC. In addition to these 6 samples (culture negative but PCR positive), both RDTs were positive for 5 additional stool specimens, of which one Crystal VC assay result was positive for both O1 and O139. The Crystal VC assay was also positive for one other study participant for only O139, but negative by all other tests, and this result was considered a false positive as no *V*. *cholerae* O139 was circulating at the time. A detailed listing of results by serogroup and serotype are included in Table 2.

**Table 2:** Comparison of RDTs with microbiological culture and PCR in 76 study participants in Bangladesh.

Microbiological Culture	Cholkit		Crystal VC		PCR	
	Positive	Negative	Positive	Negative	Positive	Negative
Culture positive (n = 19)	19	0	19	0	15	4
Culture negative (n = 57)	11	46	11	46	6	51

All PCR positive cases shown in this table were positive for *V*. *cholerae* O1 *rfb* and *ctxA* genes. All Crystal VC positive cases shown in this table were positive for *V*. *cholerae* O1. In addition to these, two Crystal VC assays were positive for O139. One was both O1 and O139 positive, counted in the table as *V*. *cholerae* O1 positive case. Another was only O139 positive (not shown in table)

### Bayesian latent class modeling

The sensitivity and specificity of all four diagnostic tests were estimated simultaneously by using Bayesian latent class modeling. In this analysis, the sensitivity of culture, PCR, Crystal VC and Cholkit were 70.8% (95% CI: 58.5–81.1), 73.6% (95% CI: 58.5–85.7), 97.5% (95% CI: 87.5–99.9) and 97.7% (95% CI: 88.4–99.9), respectively. The specificity was estimated at 99.9% (95% CI: 99.7–100) for culture, 97.2% (95% CI: 93.2–99.2) for PCR, 98.4% (95% CI: 92.0–99.9) for Crystal VC and 96.5% (95% CI: 88.6–99.6) for Cholkit (Table 3).

**Table 3:** Estimated sensitivity and specificity of four diagnostic tests for cholera in patients (95% credible intervals shown in parenthesis), using a Bayesian latent class modeling approach.

Diagnostic	Sensitivity	95% CI	Specificity	95% CI
Culture	70.8%	(58.5–81.1)	99.9%	(99.7–100)
PCR	73.6%	(58.5–85.7)	97.2%	(93.2–99.2)
Crystal VC	97.5%	(87.5–99.9)	98.4%	(92.0–99.9)
Cholkit	97.7%	(88.4–99.9)	96.5%	(88.6–99.6)

Based on data derived by performing all listed assays on each of 76 stool specimens

## Discussion

Cholera is an acute watery diarrheal disease that can be fatal if it remains undiagnosed or untreated. Rapid and accurate diagnosis of cholera at the earliest phase of an epidemic is a key feature to assist in early management of cholera outbreaks. Such early detection of a cholera outbreak is often challenging since cholera epidemics frequently occur in resource-limited areas lacking laboratory facilities and trained personnel. Here, we report development of a new rapid diagnostic test that can assist with this clinical and public health need. Cholera RDTs have a number of distinct advantages over other cholera related diagnostic options. Microbial culture is usually considered a gold standard since it is 100% specific, but this method requires at least 2–3 days in a well equipped microbiology laboratory with trained personnel, the use of selective media, and may be negative in patients who previously ingested an antimicrobial before seeking medical care, impact a vibrio specific phages, or due to delays in sample transport and handling [22–24]. In a study by Alam et al in Bangladesh that performed a detailed analysis of stool samples from patients with cholera including microbiologic culture, DFA microscopy, PCR, and phage analysis, microbiologic culturing of stool had a sensitivity of 66% [25]. In another study in India, microbiologic culturing alone of stool detected 70% of cholera cases detected by a combination of culture and molecular analysis [22]. In our current analysis, we found a sensitivity of culture alone of 70.8% (CI 58.5–81.1), a value in agreement with these previous studies, and supporting the need for additional diagnostic assays that are culture independent. Microscopic examination of fresh watery diarrheal stool using dark field microscopy can also be used to presumptively diagnose cholera [11, 26–28], although this approach requires the use of a relatively unaffordable and expensive microscope by skilled laboratory staff. The sensitivity of dark field microscopy is, unfortunately, only about 50% when compared with stool culture [27, 29]. To address these assay deficiencies, several molecular diagnostic tests have been developed. PCR assays targeting the *toxR* gene of *V*. *cholerae* or an outer membrane protein gene, *ompW*, have been used to detect the presence of *V*. *cholerae* in stool [24, 30]. Serogrouping of the strain as well as assessing for the presence of a toxigenic strain can also be determined by multiplex PCR detecting the cholera toxin gene (*ctxA*) as well as the O1 and O139-specific *rfb* genes [24, 31]. Although these techniques are more rapid than conventional culture, they require expensive reagents, modern equipment, electricity, and trained laboratory staff. Such components are usually absent from areas experiencing a cholera outbreak.

In comparison, RDTs are point-of-care tests that can be used by minimally trained staff at the bedside of a suspected cholera patient, and require no cold chain maintenance or use of advanced equipment [23]. Most RDTs for cholera detection are based on immunochromatographic or lateral flow immunoassays, targeting *V*. *cholerae* O1 and/or O139 specific antigens [32–34]. Approximately 20 cholera RDTs have been developed, and RDTs can play a critical role in the early detection and monitoring of cholera outbreaks, but standardization and reproducibility of cholera RDTs have been problematic [23]. Laboratory and field evaluation of RDTs has shown sensitivities ranging from 58 to 100%, and specificities of 60 to 100% [23, 35]. Many cholera RDTs have not been independently evaluated, and none to our knowledge have analyzed using a Bayesian latent class modeling approach to estimate true sensitivity and specificity under field conditions. To address this, we have recently developed the immunochromatographic dipstick described in this report for the rapid diagnosis of cholera. This Cholkit assay is based on the detection of *V*. *cholerae* O1 LPS, and we have evaluated its performance using a latent class modeling approach, comparing its performance to those of microbial culture, PCR (*rfb* and *ctxA* genes) analysis of stool, and use of the Crystal VC assay, a commercially available dipstick designed to detect both *V*. *cholerae* O1 and O139.

In our study of 76 cases, we found six cases that were negative by culture but positive by the other tests used, confirming the lack of sensitivity of culture also reported by others [21]. We also found 4 cases negative by PCR but positive by culture, Crystal VC and Cholkit, again suggesting false negative PCR results as reported by others [21]. Among the previous RDTs developed for the diagnosis of cholera, the Institute Pasteur (IP) dipstick has perhaps shown the most sensitivity compared with other RDTs by both laboratory and field technicians [36]. The IP dipstick technology has been transferred to a commercial company, Span Diagnostics (Surat, India) and is being produced commercially under the name of Crystal VC. This commercial version showed similar sensitivity but less specificity compared to the earlier version [37]. We used this as our RDT comparator for the current study. One of the limitations of the Crystal VC dipstick is that it may give false positive results for *V*. *cholerae* O139 [38]. Indeed, in our current study, two Crystal VC assays were positive for *V*. *cholerae* O139, although both stool specimens were negative by culture and PCR for *V*. *cholerae* O139 [10]. False positivity could lead to unfortunate and unnecessary clinical and public health responses, given the potential seriousness of missing a cholera outbreak among an at-risk population. Since O139 is not a current cause of endemic or epidemic cholera globally, we elected to focus our RDT on *V*. *cholerae* O1 alone. *V*. *cholerae* O1 can itself be characterized into Inaba and Ogawa serotypes, based on the presence of absence of a methyl group on the terminal saccharide of the O-specific polysaccharide [17, 18]. In Cholkit, we used a monoclonal antibody that recognizes both Inaba and Ogawa serotype organisms.

In our study, we used a Bayesian latent class modeling approach to estimate sensitivity and specificities of the various assay. Such an approach permits an analysis of a new diagnostic assay when a true gold standard is absent, such as is the case in cholera diagnostics [20, 21]. Our results suggest that Cholkit is highly specific, and more sensitive than culture and PCR. When considering only *V*. *cholerae* O1 results, Cholkit and Crystal VC are also highly comparable, but the probable false positivity of Crystal VC for *V*. *cholerae* O139 is disconcerting. It should be noted that our specificity analysis for Crystal VC is only based on *V*. *cholerae* O1 detection, since Cholkit was not developed to detect O139 organisms and no direct comparison for that diagnostic assay could be made. It should also be noted that positive and negative predictive values for any cholera diagnostic will reflect the current burden of disease when the assay is evaluated, and that cholera can exist in either endemic forms, or be associated with large and explosive outbreaks.

Our study has a number of limitations. First, it used a prototype assay, and the results need to be validated using a final manufactured product to assure standardization and reproducibility. We also did not assess the assay across a wide range of stool types; we focused our initial analysis on patients with acute watery diarrhea. In our study, we also analyzed the fresh stool specimens after they had been transported back to the laboratory (1–4 hours). A future analysis could perform the assay directly in the field. Also, at the time of this analysis, cholera in Dhaka was largely caused by *V*. *cholerae* O1 Inaba. The utility of the assay should also be evaluated in other outbreak settings, geographic regions, among other populations, and should include temperature and product stability analysis. Despite these limitations, we believe our results are significant. We report the development of a new and highly sensitive and specific rapid diagnostic assay for the detection of cholera cases caused by *V*. *cholerae* O1 among populations in areas lacking laboratory support and trained personnel. Early detection of such cases would assist targeted responses including diagnostic confirmation using microbiologic culturing with antimicrobial resistance profiling and initiation of cholera treatment and control efforts.
